# Supplementary material for: An Improved In Vitro Blood-Brain Barrier Model for the Evaluation of Drug Permeability Using Transwell with Shear Stress
Source: Pharmaceutics. 2023 Dec 28;16(1):48. doi: 10.3390/pharmaceutics16010048 (PMC10820479; doi:10.3390/pharmaceutics16010048)
Supplement: Supplementary file 1 [file pharmaceutics-16-00048-s001.zip › pharmaceutics-2791761-supplementary.pdf]

Table S1. The PCR primer sequences

Gene	Forward primer (5′-3′)	Reverse primer (5′-3′)	Ref
CLDN5 (Claudin-5)	GTGCTACACCCAGTGTGCTG	CCAGTTCAGGTGACACCACTT	[1]
OCLN (Occludin)	AAGGTCAAAGAGAACAGAGCAAGA	TATTCCCTGATCCAGTCCTCCTC	[2]
CDH5 (VE-cadherin)	AATGCGTCCGTGCCTGAGTCGT	GTGGTCTCCCACAGTGGGGTCG	[1]
GAPDH	CAACTACTGGTTTACATGTTC	GCCAGTGGACTCCACGAC	[3]

Table S2. The LC-MS/MS condition 1

Instrument	LC<br>MS/MS	Agilent 1260 binary HPLC system<br>Agilent 6460 Triple-quadruple mass spectrometer		
Column	Agilent Poroshell 120 EC-C18 (3.0 × 50 mm, 2.7 μm)			
Mobile phase	A : 5 mM Ammonium formate in water (pH 4)<br>B : MeOH			
Flow rate	0.3 mL/min			
Column oven temperature	40℃			
Injection volume	5 μL			
MRM condition	m/z	Fragmentor (V)	Collison Energy (V)	Polarity
Atenolol	267→145	123	24	(+)
Dantrolene	313→200	100	10	(-)
Naproxen	231.1→185.1	75	6	(+)
Phenytoin	253→182	100	12	(+)

Table S3. The LC-MS/MS condition 2

Instrument	LC	Shimadzu UFLC XR instrument
	MS/MS	TSQ Quantum Ultra triple quadrupole mass spectrometers
Column	YMC triart C18 (2.0 × 50 mm, 3 μm)	
Mobile phase	A : 5 mM Ammonium formate in water (pH 4)<br>B : MeOH	
Flow rate	0.3 mL/min	
Column oven temperature	40℃	
Injection volume	10 μL	

MRM condition	m/z	Collison Energy (V)	T Lens (V)	Polarity
Carbamazepine	237.2→194.1	19	122	(+)
Donepezil	380.3→91.1	35	95	(+)
Fexofenadine	502.2→466.4	25	130	(+)
Metoprolol	268.2→133.1	25	135	(+)
Midazolam	326.1→291.3	25	142	(+)
Propranolol	260→183.1	17	163	(+)
Sulpiride	342.3→112	25	155	(+)

1. Li, N.; Sui, Z.; Liu, Y.; Wang, D.; Ge, G.; Yang, L. A fast screening model for drug permeability assessment based on native small intestinal extracellular matrix. *RSC Adv* **2018**, *8*, 34514-34524, doi:10.1039/c8ra05992f.
2. Ito, R.; Morio, H.; Baba, T.; Sakaguchi, Y.; Wakayama, N.; Isogai, R.; Yamaura, Y.; Komori, T.; Furihata, T. In Vitro-In Vivo Correlation of Blood-Brain Barrier Permeability of Drugs: A Feasibility Study Towards Development of Prediction Methods for Brain Drug Concentration in Humans. *Pharm Res* **2022**, *39*, 1575-1586, doi:10.1007/s11095-022-03189-y.
3. Cai, B.; Miao, Y.; Liu, Y.; Xu, X.; Guan, S.; Wu, J.; Liu, Y. Nuclear multidrug-resistance related protein 1 contributes to multidrug-resistance of mucoepidermoid carcinoma mainly via regulating multidrug-resistance protein 1: a human mucoepidermoid carcinoma cells model and Spearman's rank correlation analysis. *PLoS One* **2013**, *8*, e69611, doi:10.1371/journal.pone.0069611.
